# Polydopamine‐Wool Textiles for Sustainable Interfacial Solar Vapor Generation

**DOI:** 10.1002/advs.77609

**Published:** 2026-09-06

**Authors:** Kyuin Park, Jintong Gao, Hansadi Jayamaha, Yipu Wang, Mia Bressler, Lenan Zhang, Larissa M. Shepherd

**Affiliations:** ^1^ Department of Human Centered Design Cornell Human Ecology Cornell University Ithaca New York USA; ^2^ Sibley School of Mechanical and Aerospace Engineering Cornell Duffield Engineering Cornell University Ithaca New York USA; ^3^ School of Civil and Environmental Engineering Cornell Duffield Engineering Cornell University Ithaca New York USA

**Keywords:** functional textiles, nanostructures, polydopamine, solar desalination, textiles, ultrablack, vapor generation

## Abstract

Interfacial solar vapor generation (ISVG), which harnesses sunlight for water distillation, presents a promising strategy to address global water scarcity. While conventional 3D evaporators offer high evaporation performance, their widespread implementation remains constrained by material selection, cost, and fabrication methods. Herein, we present a nature‐inspired, polydopamine (PDA)‐dyed wool textile as a 2.5D evaporator for ISVG, leveraging its double‐sided evaporating surfaces to maximize overall evaporation performance. The inherent fibrous architecture of wool textiles enables efficient water transport via natural capillary and exhibits effective solar absorption through photothermal PDA dyeing. The hierarchical nanostructure on micro‐scale fibers, created by subsequent plasma etching, further amplifies the light trapping and moisture wicking capabilities. Under 1‐Sun illumination, the PDA‐wool and ultrablack PDA‐wool textiles achieve evaporation rates of 2.21 and 2.43 kg/m^2^/h, respectively. In modeled calculations, both outperform the commercial baseline, with ultrablack‐wool exceling in low‐solar regions. The all‐natural composition eliminates concerns regarding the leaching of microplastics or hazardous nanomaterials. Furthermore, the system maintains a stable evaporation rate for 10 h of continuous operation without salt‐accumulation. This approach opens new opportunities for natural textiles and offers a cost‐effective route toward deployment of textile‐based solar‐driven water purification systems with strong practical viability in off‐grid and socially vulnerable communities.

## Graphical abstract

A natural wool textile engineered for high solar absorbance and a fast‐wicking structure serves as a highly efficient, single‐layer system for interfacial solar vapor generation (ISVG). This integrated evaporator seamlessly couples rapid water transport with continuous evaporation. Ultimately, this sustainable photothermal design offers a scalable, eco‐friendly approach to advance clean water production.

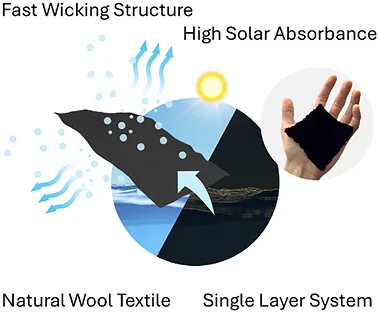

## 1 Introduction

Driven by climate change and rapid urbanization, global demand for freshwater is increasingly outpacing available resources in both urban and rural areas [1, 2]. By 2050, water scarcity is projected to affect nearly half of the global urban population, a 50% rise since 2016 [3, 4, 5, 6]. The crisis extends beyond quantity, with the report showing that only 132 of 197 countries maintain metric standards for drinkable water, leaving roughly one‐third of the global population without regulated water quality protections [7, 8]. Conventional desalination approaches such as reverse osmosis and thermal distillation are effective, but infrastructure‐heavy and energy‐intensive, driving up costs and greenhouse gas emissions in ways that exclude economically and socially disadvantaged communities even in major cities [9, 10, 11, 12, 13, 14, 15, 16].

Interfacial solar vapor generation (ISVG) has emerged as a promising lower‐barrier alternative, concentrating solar energy at the air‐water interface rather than heating the entire bulk liquid [9, 12, 17, 18, 19, 20, 21, 22, 23, 24, 25]. Recent work has shifted toward 3D evaporator architectures (e.g., foam, conical, cylindrical, and vertical geometries), which outperform flat 2D systems by expanding the air‐water interfacial area, harvesting environmental energy, and minimizing reflective losses, pushing evaporation rates well beyond the ∼1.5 kg/m^2^/h ceiling [25, 26, 27, 28, 29, 30]. An intermediate 2.5D configuration, in which a planar textile exposes both its front and back sides to the environment, is referred to as two‐sided evaporation (2E) vs. single‐sided evaporation (1E), and offers a practical bridge between simple flat evaporators and fully volumetric 3D architectures [31]. Despite these advances, translating lab‐scale evaporators into practical devices remains challenging. Common barriers include complex fabrication, the risk of microplastic and nanomaterial leaching, salt fouling, and limited scalability [13, 14, 32, 33, 34, 35].

These constraints have driven growing interest in natural, sustainable textile scaffolds, whose fibrous capillary networks passively transport water while their inherent porosity allows vapor to escape readily [17, 21, 30, 34, 36, 37, 38, 39]. Cotton has been the most widely studied textile due to its low cost and hydrophilicity [40], with representative coatings such as carbon black on cotton‐basalt blends and biomimetic polypyrrole yielding evaporation rates of 1.52 and 2.02 kg/m^2^/h, respectively [41, 42]. Achieving high‐performance ISVG, however, typically requires either intricate bottom‐up yarn fabrication or synthetic nanomaterials such as carbon nanotubes, graphene oxide (GO), reduced‐GO sheets, or silver nanoparticles, compromising scalability and raising environmental concerns [43].

Natural wool presents an underexplored alternative. As a renewable, biodegradable protein fiber harvested annually from sheep, it generates no microplastic byproducts [44]. Its inherent crimp, scale, and low thermal conductivity (0.043–0.046 W/mK) help localize heat at the evaporation surface [45, 46], while its strong moisture‐wicking property supports continuous water transport [44]. Because wool on its own does not possess the necessary photothermal functionality, we explore the incorporation of polydopamine (PDA) as a biocompatible and biodegradable photothermal agent [47]. Mussel‐protein‐inspired PDA, with its catechol and amine functionalities, interacts with wool fibers during in situ polymerization [48, 49, 50], simultaneously imparting broadband photothermal conversion [51, 52], UV protection [51], flame retardancy [53], and antimicrobial and anti‐fouling [54] properties. Furthermore, this integrated PDA‐wool network serves as a substrate for subsequent plasma etching, enabling a light‐trapping hierarchical nanostructured surface [50].

In this work, we present a scalable, textile‐based evaporator combining natural wool with PDA functionalization for sustainable ISVG. We evaluate three distinct wool textile architectures—single jersey, double knit (interlock), and flannel. A baseline PDA dyeing process imparts reliable photothermal activity, while a subsequent plasma etching step pushes solar absorbance to an ultrablack state (< 0.5% reflectance) [50, 55], yielding a stepwise increase in evaporation rate. The benefit of the 2.5D orientation is reflected in both illuminated and dark evaporation performance. Under 1‐Sun illumination, the best‐performing ultrablack wool textile achieves 2.43 kg/m^2^/h in the 2E orientation compared to 1.39 kg/m^2^/h in the 1E orientation, while dark evaporation increases nearly five‐fold from ∼0.2 to ∼0.95 kg/m^2^/h upon switching from 1E to 2E. Our system shows no salt accumulation over 10‐h continuous operation and produces distillate that exceeds WHO and EPA standards for drinkable water. Next, we compare the wide‐angle solar absorbance of PDA‐dyed and ultrablack wool to commercial wool and demonstrate how our water‐generation method could practically benefit economically and socially vulnerable populations in both coastal and inland areas by estimating their annual vapor production. Finally, the outdoor field test using a 10 wt.% NaCl solution successfully demonstrates the real‐world applicability. Our research demonstrates that a natural fiber‐based evaporator can achieve high performance without the scalability and toxicity trade‐offs of synthetic nanomaterial approaches.

## 2 Results and Discussion

### 2.1 Optical and Material Properties of Wool Textiles

**FIGURE 1:**
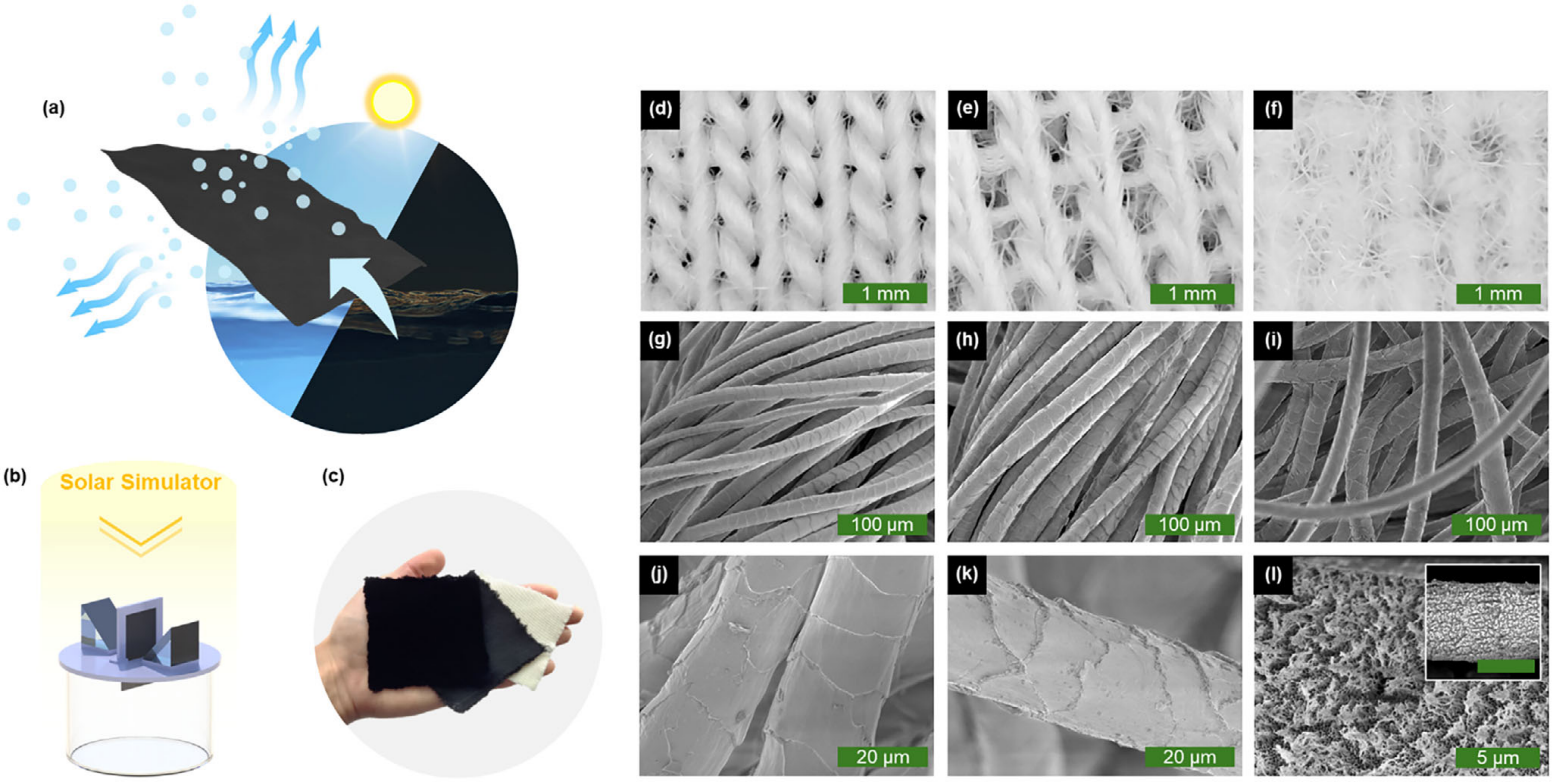
(a) Schematic of the all‐day evaporator textile for interfacial solar vapor generation. (b) Experimental setup utilizing a solar simulator. (c) Photographs of Ultrablack (UB), PDA‐dyed (P), and undyed white (W) double‐knit (D) wool textile swatches. (d–f) Optical microscope images of (d) single‐jersey (S), (e) double‐knit (D), and (f) flannel (F) wool fabrics. (g–l) SEM images of (g) WS, (h) WD, (i) WF, (j) WF (zoomed‐in), (k) PF, and (l) UBD. Scale bars: 1 mm (d–f), 100 µm (g–i), 20 µm (j–l). Inset scale bar: 20 µm (l).

Figure 1a illustrates the proposed textile ISVG evaporator, integrating solar‐absorbing and moisture‐wicking capabilities to sustain continuous vapor generation both day and night. To evaluate the advantage of two‐sided evaporation (2E), a vertical orientation was tested against the traditional 2D, one‐sided (1E), setup (Figure 1b). In this vertical configuration, one or two mirrors are positioned to simulate the additional solar irradiance reflected from the surrounding environment, allowing us to isolate this effect without altering the intensity of the solar simulator. In subsequent sections, we narrow our evaluations to a 1‐Sun (1S) orientation to provide a more realistic assessment. This approach aligns with other vertical 2.5D solar‐facing studies [31, 56, 57], which utilize two evaporating surfaces and a back surface that captures reflected light to enhance evaporation rates. For comparison, we evaluate a 2‐Sun (2S) scenario with mirrors to demonstrate the maximum potential of surrounding reflectance. While such direct and abundant supply of reflected light would be unrealistic, the 2S setup simulates a theoretical limit where the back of the sun‐facing textile receives illumination equal to the direct sunlight. Figure 1c highlights the visual evolution, simplicity, and portability of these eco‐friendly evaporators. We start with pristine white (W) wool textiles, which are then dyed in polydopamine (PDA) to create PDA‐dyed (P) wool. The scalability of the dyeing process has been demonstrated (Figure S1). A subsequent plasma etching step transforms the material into ultrablack (UB) wool, achieving an extreme dark state (<0.5% reflectance) [50].

Using optical and scanning electron microscopy (SEM) we characterize the structural variations among the three textile architectures, single jersey (S), double knit (interlock) (D), and flannel (F), and to observe their morphological evolution from pristine white (W), to PDA‐dyed (P), and finally to the ultrablack state (UB, Figure 1d–l). While S and D are both knitted structures, D (Figure 1e) exhibits a significantly looser, more open macroscopic structure compared to S (Figure 1d). Conversely, F (Figure 1f) is a woven twill textile characterized by a densely napped, fibrous surface texture. High‐magnification SEM images of the pristine white textiles reveal minimal variations in individual fiber diameter and inherent surface roughness (Figure 1g–i). On the contrary, while F is a woven textile, its surface displays a random, non‐woven‐like network of fibers due to the napping of the underlying textile structure (Figure 1i). Following PDA‐dyeing, all textile fibers exhibit a uniform coating (Figure 1k). As demonstrated in our prior work [50], PDA deeply penetrates the wool fibers [49], ensuring that the subsequent plasma etching step does not simply strip away the coating to reveal a white interior. Instead, the etching process generates hierarchical light‐trapping nanostructures [58, 59]. The synergistic effect between this retained deep internal coloration and the newly formed bundles of nanofibrils (Figure 1l) fundamentally enables the extreme ultrablack appearance (Figure S2).

**FIGURE 2:**
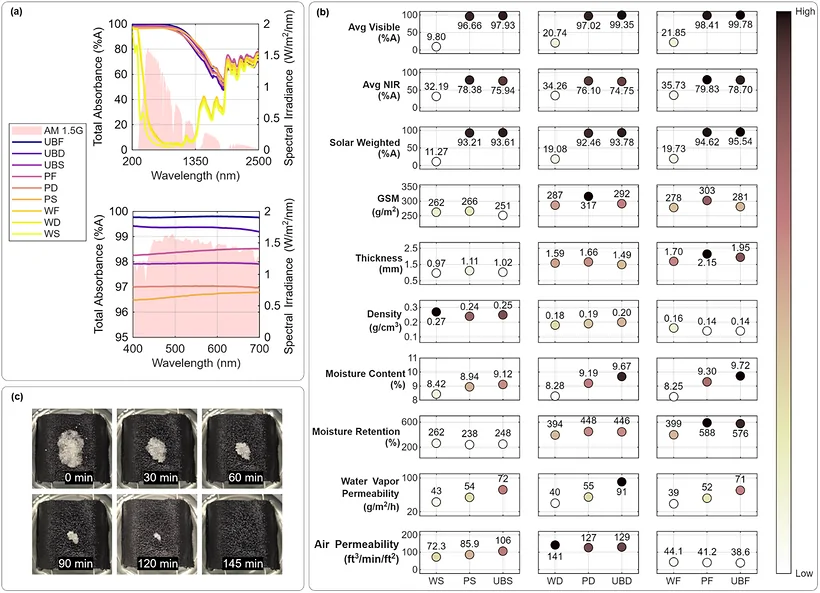
(a) Total absorbance of ultrablack flannel (UBF), ultrablack double‐knit (UBD), ultrablack single jersey (UBS), PDA‐dyed flannel (PF), PD, PS, white undyed flannel (WF), WD, and WS across the full solar spectrum (top) and the visible region (bottom). (b) Comparison of material properties among the samples. (c) Photographs demonstrating the time‐dependent salt dissolution.

The ultraviolet (UV, 280–400 nm) and near‐infrared (NIR, 700–2500 nm) absorbance capabilities of PDA [49, 60] are evident across all treated samples (PS, PD, PF, UBS, UBD, and UBF), as shown in Figure 2a. In the visible spectrum (400–700 nm), the UBF, which is plasma‐etched on both sides, exhibits the highest optical performance with an exceptional 99.78% total absorbance (A). This value is calculated via A = 1 – (R + T), where R and T represent total measured reflectance and transmittance, respectively (Figure S3). UBF is closely followed by UBD and PF. Notably, PF demonstrates higher absorbance than UBS, which is an expected consequence of the naturally open knit structure of the S fabrics, which allows for higher transmittance (Figure 1d–f). Measured transmittance and visual comparison between WS, WD, and WF prove the highest transmittance of S, followed by D, and F (Figures S3 and S4). Upon evaluation of mid‐IR (2.5–20 µm) emissivity, neither PDA‐dyed nor the UB‐treated samples exhibit a meaningful difference from their undyed counterparts, which already behave as near‐blackbody emitters in the mid‐IR region (Figure S5).

Figure 2b provides a comprehensive summary of how the P and UB treatments influence textile material properties, including optical performance, areal weight (GSM, g/m^2^), thickness (mm), density (g/cm^3^), moisture content (%), moisture retention (%), water vapor permeability (g/m^2^/h), and air permeability (ft^3^/min/ft^2^). Across all architectures of wool, the initial PDA‐dyeing (PS, PD, PF) significantly increases the %A in both visible and NIR spectra. The UB treatment yields a further, stepwise amplification of visible absorbance (Figure 2a). In NIR, however, a slight decrease is observed. This minor reduction, alongside corresponding slight decreases in GSM and thickness, is a direct result of the nature of the plasma etching, which affects the outermost layer of the fabric. Because this etching is highly localized to the surface, the overall bulk density of the UB textiles remains nearly identical to their P counterparts. Any minor fluctuations in density or GSM across the samples can be attributed not only to PDA deposition, but also to the structural swelling and shrinking inherent to the wet processing and drying phases of fabrication.

To evaluate the photothermal potential of these materials, the average solar‐weighted absorbance (SWA) is calculated using Equation (1):

(1) $$SWA=\frac{\int_{280}^{2500}A\left(\lambda \right)I_{solar}\left(\lambda \right)d\lambda }{\int_{280}^{2500}I_{solar}\left(\lambda \right)d\lambda }$$

where *A*(*λ*) is the total absorbance at a specific wavelength (*λ*) and *I_solar_* (*λ*) is the spectral irradiance (W/m^2^/nm) based on the AM1.5G standard. The average SWA is maintained above 92% for all P and UB textiles, with UBF peaking at 95.54%, followed by PF at 94.62%. In comparison to the regular total absorbance, the SWA metric underscores the critical importance of maximizing absorption in the visible range, where the solar spectral energy is most intense.

Beyond optical performance, an efficient ISVG evaporator requires highly optimized fluid transport. To assess this, standardized measurements of moisture content (MC), moisture retention (MR), water vapor permeability (WVP), and air permeability (AP) are used. We observe a progressive increase in MC from the W to P and to UB variants (Equation (2) in the Experimental Section). This enhancement is driven by synergistic chemical and structural modifications of the fiber surface. Initially, PDA‐dyeing introduces a high density of hydrophilic catechol and amine groups, facilitating abundant hydrogen bonding with water molecules. Subsequent air plasma etching (UB treatment) further amplifies moisture absorption capacity by increasing oxygen‐ and nitrogen‐containing species via surface oxidation, while simultaneously inducing hierarchical nanoscale roughness. This etched topography increases the effective surface area for capillary wicking. Interestingly, MR (wet pick‐up capacity) remained largely unchanged after plasma etching (Equation (3) in the Experimental Section). We attribute the overall capacity to hold bulk fluid remaining stable to the plasma only affecting the outermost surface of fibers without alteration of the underlying bulk yarn structure.

Finally, analyzing vapor and air transport reveals further functional benefits of PDA‐dyeing and UB treatment. AP measures the bulk volume of air forced through the fabric under pressure, whereas WVP measures the unpressurized transmission of evaporated vapor. While no significant trend was apparent in the AP data, WVP steadily increased for P samples and further increased for the UB samples (Equation (4) in the Experimental Section). WVP tracks the mass change of the bulk water beneath the textile; therefore, these incremental changes suggest that the chemically and structurally modified textiles actively absorb and transport moisture to the evaporative surface, rather than simply allowing vapor to passively bypass through the inherent fabric pores.

Figure 2c provides the salt dissolution capability of textiles. As salt crystallization blocks capillary wicking channels and severely scatters incoming light, preventing salt accumulation is a critical challenge in ISVG. To evaluate natural salt dissolution and rejection, a concentrated salt mound is placed directly on the surface of the UBD sample during continuous wicking of 10 wt.% NaCl solution [61, 62]. Over time, the salt progressively dissolves and is transported back into the underlying bulk water, completely disappearing within a 145‐min timeframe (Figure S6). This convection‐diffusion backflow effect and gravitational dissolution [12] demonstrate the efficacy of the fabric's wicking structure, driven by continuous capillary fluid pumping and concentration‐gradient‐driven diffusion (Video S1), ensuring long‐term efficiency and operational stability.

### 2.2 Interfacial Solar Vapor Generation/Desalination Performance

**FIGURE 3:**
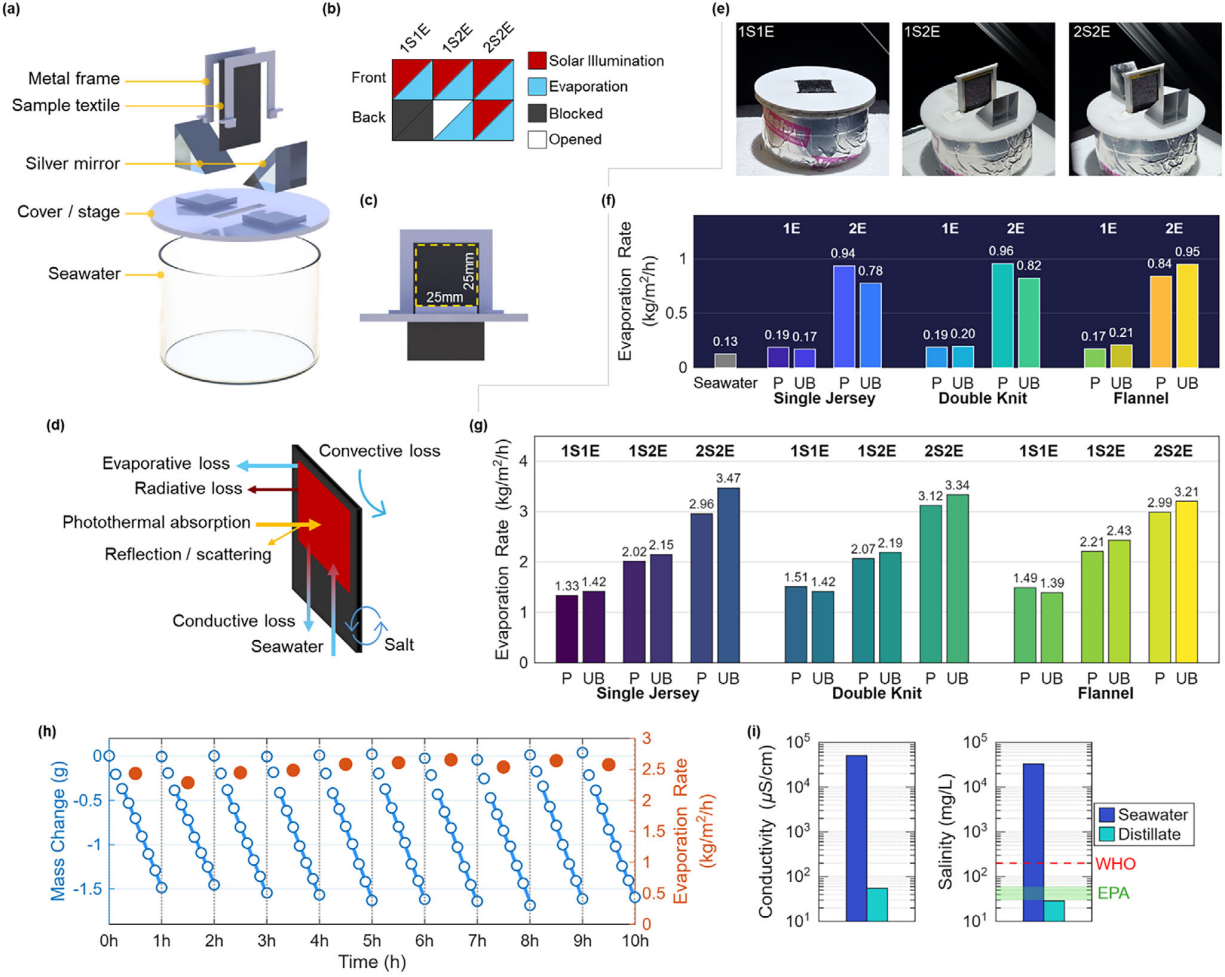
(a) Expanded schematic of the experimental setup. (b) Testing configurations and their corresponding abbreviations. (c) Textile orientation and illumination area during vertical two‐sided evaporation (2E). (d) Energy balance and liquid transport mechanisms governing the evaporation rate. (e) Photographs of the physical setups. (f) Dark evaporation rates under horizontal one‐sided (1E) and vertical two‐sided (2E) conditions. (g) Total evaporation rates of the samples. (h) Continuous 10‐h stability of UBF sample. (i) Purity test before and after distillation of seawater.

To quantify evaporation performance and understand the impact of the photothermal treatments across the three distinct textile architectures, we systematically evaluate the samples under three different configurations (Figure 3). Textiles inherently possess exceptional wicking capabilities with their hierarchical structure comprising bundles of fibers twisted into yarns which are then integrated into a bulk textile. This creates robust capillary networks for efficient liquid transport. When oriented vertically, these textiles function as self‐sufficient ISVG evaporators without requiring any external pumping mechanisms or secondary wicking layers. As illustrated in Figure 3a, one edge of the textile (<2 cm) was submerged into the bulk seawater through a narrow slit in the stage to draw seawater continuously. A major advantage of this vertically oriented, textile‐based ISVG is its ability to utilize both the front and back surfaces, functioning effectively as a 2.5D or 3D evaporator [31, 56, 63, 64, 65]. To isolate and compare the specific contributions of increased evaporative surface area vs. increased solar energy input, three testing conditions are established (Figure 3b): a traditional horizontal, one‐sided evaporation setup (1S1E); a vertical, two‐sided evaporation setup under 1‐Sun illumination (1000 W/m^2^, 1S2E); and a vertical, two‐sided evaporation setup utilizing rear mirrors to achieve 2‐Sun equivalent illumination (2000 W/m^2^, 2S2E). As noted earlier, the 1S2E orientation simulates a realistic scenario where the front of the textile directly faces the sun. In contrast, the 2S2E orientation represents a near‐impossible maximum condition, where the back surface receives identical illumination to the front without the use of mirrors.

To ensure accuracy of our measurements, stringent conditions are applied. The area of solar illumination for 1S2E and 2S2E is restricted to be ∼1 mm smaller than the internal dimensions of the metal mounting frame, and the outer edges of the fabric are completely masked with the frame and aluminum tape to prevent unintended solar absorption or peripheral evaporation (Figure 3c). By strictly eliminating the edge‐effect, a common artifact in lab‐scale testing that could potentially inflate evaporation rates by utilizing the unilluminated perimeter of a small sample, we ensured that our thermal energy transfer analysis (Figure 3d) focuses solely on the primary faces of the textile. In contrast, traditional 1S1E setups are unframed, allowing the edges to be simply exposed. Because our study focuses on the significant benefits of the 2E orientation and its synergistic effect with the textile‐based materials, it is important to note that the 1E setup possessed an artificial advantage during measurement due to this uninhibited edge evaporation. Consequently, the higher evaporation rate observed for the fully masked 2E setup underscores its true efficiency, as it outperformed the 1E configuration despite this built‐in disadvantage.

We first establish the baseline dark evaporation rates (Figure 3f) using bulk seawater. By transitioning from the horizontal 1E condition to the vertical 2E orientation, dark evaporation rates increase nearly five‐fold, jumping from ∼0.2 to ∼0.95 kg/m^2^/h. Interestingly, we observe a slight decrease in the dark evaporation rate for the UBS and UBD samples in the 2E orientation compared to their PDA‐dyed counterparts. As previously noted (Figure 2b), the S and D architectures are structurally similar, though D possesses a larger, more open structure with lower density. Because the UB plasma treatment significantly increases both the surface area and the chemical hydrophilicity of the fibers, the capillary force and surface tension within these relatively dense, flat knits may bind the water molecules more strongly, slightly retarding spontaneous dark evaporation. In contrast, the flannel (F) textiles, characterized by their loosely napped hairy surface fibers atop a woven core structure and low density (Figures 1f,i, 2d, and Figure S4), consistently facilitate easier moisture vaporization into the ambient air from both surfaces. Notably, the observed evaporation rates do not strictly correlate with WVP or AP trends (Figure 2b). This difference in trends arises because WVP and AP standard protocols quantify the vapor and air transmission through completely dry textiles, a fundamentally different condition than the evaporation from water‐saturated textiles evaluated in this section.

Under a solar simulator (Figure 3g), the traditional 2D (1S1E) configuration yields evaporation rates of 1.33–1.51 kg/m^2^/h. Transitioning to 2.5D vertical orientation (1S2E), however, dramatically amplifies performance, with PF and UBF achieving evaporation rates of 2.21 and 2.43 kg/m^2^/h, respectively. The boosted evaporation rates under both dark and 1‐Sun conditions across all textile structures underscore the significant potential of dual‐surface ISVG evaporators. By leveraging a thin, lightweight form factor, these systems maximize the air‐water interface, effectively amplifying the evaporation of intermediate water molecules. To push the performance limits by simulating a condition with maximum reflections from the surrounding environment, we introduce the 2S2E configuration. Rather than increasing the intensity or utilizing an optical concentrator (e.g., Fresnel lens) to increase incident intensity on a single side, we position a mirror to illuminate the reverse side of the existing fabric. This spatial configuration effectively achieves 2‐Sun energy input while maintaining the exact same material footprint, maximizing the utility of the photothermal textile. Under this 2S2E condition, UBS exhibits the most significant performance leap, reaching 3.47 kg/m^2^/h. UBD and UBF also demonstrate significant increases, reaching 3.34 and 3.21 kg/m^2^/h, respectively, with all PDA‐dyed variants surpassing 2.95 kg/m^2^/h (Table S1). When evaluating solar‐to‐vapor efficiency, it is important to note that the 2S2E configuration in our study doubles the solar input by utilizing two mirrors. Consequently, unless the evaporation rate also doubles from 1S2E, the calculated efficiency decreases. With further optimization of the overall system and design, if the back surface relies solely on ambient reflected light (as simulated by our 2S2E orientation), this illumination is excluded from the direct solar input. This allows the system to boost the overall evaporation rate without penalizing calculated efficiency, aligning with strategies established in previous 2.5D studies [31, 56, 57].

To investigate environmental impacts, we conduct COMSOL simulations varying solar irradiance, convection, and surrounding ambient temperature (Note S1). Although the models assume a simplified, non‐porous, and flat surface, the resulting trends align closely with our experimental data (Table S3). Here, the input solar‐weighted‐absorbance (SWA) of PF and UBF are 0.9462 and 0.9554, respectively. A commercial black wool flannel (CF) is introduced as a comparison, which has an SWA of 0.7179. The simulations reveal that 2E orientation maintains a lower surface temperature than 1E, drawing more radiative and convective heat from the environment, resulting in a higher evaporation rate. By aligning simulated dark evaporation rates with our experimental results, we determine that shifting from a 1E to a 2E orientation provides an evaporation boost equivalent to increasing natural convection by 0.06 to 0.2 m/s on the simplified flat material. (Figures S7–S9) We found that 2E configurations are significantly more responsive to the environmental effect when 1E evaporation improves only marginally. Furthermore, high‐absorbance textiles (PF and UBF) consistently outperform the commercial option (CF), particularly under higher total solar inputs where the reliance on natural convection diminishes (Note S1 and Figure S9). Ultimately, these results confirm the synergistic performance enhancement achieved by combining a 2.5D (2E) configuration and high solar absorbance.

Lastly, we evaluate the stability and desalination efficacy of our system using the UBF sample in the 1S2E orientation. We expose the system to continuous 1‐Sun illumination for 10 h using bulk seawater. Not only does the evaporation rate remain entirely stable throughout the duration at ∼2.5 kg/m^2^/h (Figure 3h and Figure S10), but visual inspection of the retrieved textile confirms no salt crystallization on either side of the textile. This corroborates that the capillary force, hydrophilic liquid transport, and gravitational salt dissolution of the textile structure continue to outpace the evaporation rate, suppressing salt accumulation (salt reflux capability under dark conditions in Figure 2c and Video S1). In addition, accelerated 42‐h UV exposure (equivalent to 9–11 days) causes no significant changes in color or durability (Note S2, Figures S11 and S12, and Tables S4 and S5).

To assess the water quality, we collect and analyze the distillate (Figure 3i). While the initial bulk seawater registers a conductivity of 50574 µS/cm and salinity of 32.96 ppt (parts per thousand) (equivalent to 32960 mg/L), the collected distillate exhibits a conductivity of 55.071 µS/cm, which was calculated to a salinity of 28.8 mg/L (0.0288 ppt), which meets the sodium level for all reported countries (Equation (6) in Experimental Section) [8]. We further investigate water quality by calculating the salt rejection ratio (*R*) (Equation (7) in the Experimental Section) of the PDA‐wool evaporator, which was found to be ∼99.9%. For context, the World Health Organization (WHO) guideline for sodium in drinking‐water is about 200 mg/L [7], a standard our system comfortably exceeds (Figure 3i). Furthermore, the US Environmental Protection Agency (EPA) notes that the threshold for human taste detection of salt begins between 30 and 60 mg/L [66], meaning the water we generate by our textile evaporator is not only pure but also highly palatable. While this study does not focus on the system design and the distillate collection mechanism, future refinements of these components are expected to yield further improvements in the final distillate quality.

### 2.3 Angular Independence and Modeled Performance

**FIGURE 4:**
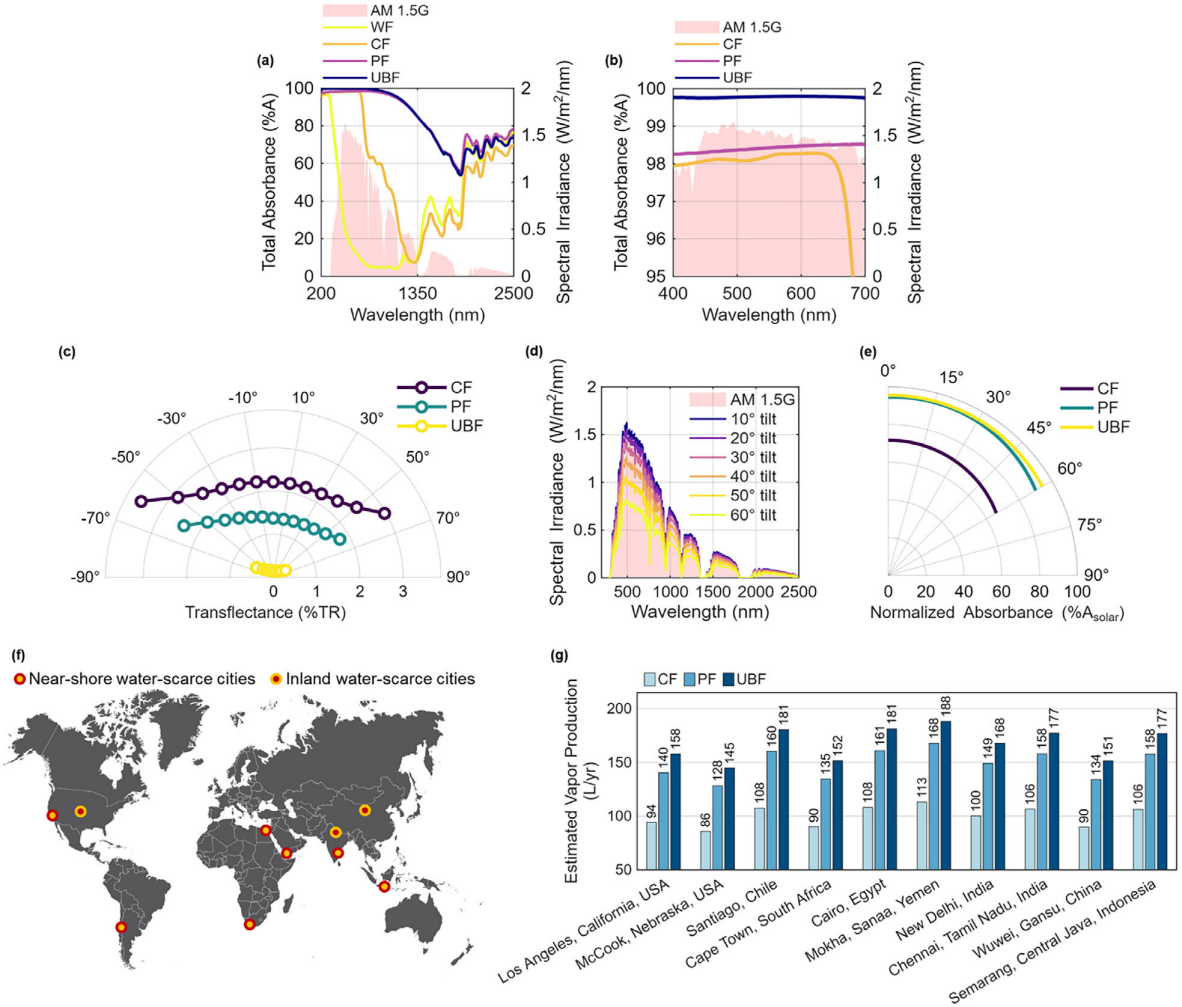
(a) Total absorbance of flannel (F) samples against the commercially available black flannel (CF) across the full solar spectrum. (b) Total absorbance of CF, PF, and UBF across the visible spectrum. (c) Average visible transflectance of the F textiles at tilt angles ranging from −60° to +60°. (d) Spectral irradiance of the AM1.5G spectrum illustrating Lambert's cosine law. (e) Normalized full‐spectrum absorbance derived from transflectance measurements, weighted according to the decrease in spectral irradiance in (d). (f) World map with large and small, near‐shore and inland cities facing water scarcity [3, 67]. (g) Estimated annual daytime vapor production for the F textiles at various geographic locations indicated in (f), calculated using Global Horizontal Irradiance (GHI) and solar zenith angle data from the National Solar Radiation Database (NSRDB) [68].

To contextualize this optical and vapor generation performance against conventional materials, a commercially available, black‐colored wool flannel (CF) is introduced as a reference (Figure 4). Flannel was chosen for this assessment because PF and UBF exhibited the highest solar‐weighted absorbance, moisture content, and moisture retention, and performed best under the most practical 1S2E condition. This comparison preserves structural similarity in wicking and interfacial evaporation mechanisms, isolating photothermal efficacy as the key variable between CF, PF, and UBF. Figure 4a compares full solar spectrum absorbance across the three flannel variants. Although CF and PF appear visually identical (Figure S13), CF's non‐photothermal dye absorbs minimally in the NIR region. In the visible range alone (Figure 4b), CF and PF show nearly identical absorbance, but CF exhibits a sharp drop beyond ∼650 nm, extending into low absorbance across the NIR.

In real‐world applications, the angle of incoming sunlight shifts continuously throughout the day and across seasons. To evaluate optical performance under these dynamic conditions, transflectance (TR) was measured at varying incident angles using a center‐mounted integrating sphere to quantify both reflected and transmitted light (Figure 4c). Increasing, or shifting, the incident angle from 0° produces two primary optical effects: transmittance decreases due to the extended optical path length through the textile, and reflectance increases at high angles. This high‐angle specular reflectance arises from Fresnel reflection coupled with topographical shadowing, where superficial fibers progressively obscure the inter‐fiber voids visible at normal incidence. Despite their similar visible absorbance (Figure 4b), CF and PF diverge at higher angles: CF shows up to ∼3.5%TR, approximately 0.8–1.2% higher than PF. UBF remains well below both, reaching a maximum of only ∼0.45%TR at −60° and a lowest of ∼0.15%TR at 0°, confirming that its hierarchical nanostructure maintains an ultrablack state even at high tilt angles.

This angular stability is particularly relevant given that the sun rarely occupies the precise zenith (0°) during a year. The angular transflectance data are used to calculate total solar spectrum absorbance by accounting for the reduction in solar irradiance described by Lambert's cosine law (Figure 4d). Figure 4e presents the normalized full solar spectrum absorbance as a function of incident angle. At 0°, the solar‐weighted average absorbances (SWA) for CF, PF, and UBF are 71.79%, 94.62%, and 95.54%, respectively (Figure 2b and Figure S14). At 60° tilt, these values decrease to 65.83%, 89.80%, and 94.10%, corresponding to drops of 8.30%, 5.09%, and 1.51%, respectively, with respect to 0°. Notably, this wide‐angle solar absorbance provides a significant practical advantage by reducing the ISVG system's need to physically reorient or track the sun. Instead, a horizontally positioned UBF with both surfaces exposed can maintain high solar absorbance even at high zenith angles, ensuring consistent evaporation throughout the day and across seasons.

To translate these properties into practical geographic context, annual vapor production is modeled across varying latitudes and weather profiles (Figure 4f,g). Measured evaporation rate data (Figure 3g and Table S1) and the normalized absorbance (Figure 4e) are integrated with Global Horizontal Irradiance (GHI) data and solar zenith angle from the National Solar Radiation Database (NSRDB) [68]. The GHI dataset provides both hourly average irradiance (accounting for diffuse light and cloud cover) alongside the corresponding solar zenith angle over a 365‐day period. Angle‐dependent absorbance is calculated for each hour using polynomial fits derived from our angular transflectance data and multiplied by the hourly GHI to estimate total absorbed solar energy in multiple parts of the world indicated as water‐scarce cities, despite the population and location [3, 67]. While inland regions inherently lack seawater resources, the performance metrics established in this study use seawater as a standardized benchmark to demonstrate the PDA‐wool textile‐based ISVG's broader potential for treating groundwater and wastewater. The model in Figure 4f,g assumes a 2E orientation with dynamic solar input, but in horizontal geometry to simplify the effect of incoming solar irradiance across hours and seasons, with solar absorbance assumed to decrease linearly to 0% at 90° for zenith angles beyond 60°. Assuming a horizontal orientation prevents the overestimation associated with idealized models, such as systems that continuously track the sun or perfectly align with peak daily sunlight. Instead, this static horizontal baseline ensures a more conservative and realistic projection of annual vapor production across diverse geographical regions. Table S6 provides the calculated numerical values for the estimated annual vapor production shown in Figure 4g. (Raw 24‐h (Day1) data from Santiago, Chile for CF, PF, and UBF are shown in Tables S7–S9, respectively, as an example of how the solar zenith angle, GHI, and experimental data were utilized.)

The geographic modeling reveals notable trends. In Figure 4g, the calculations utilize 20 × 20 cm samples of CF, PF, and UBF, representing an area just larger than an adult male's hand. Despite modest differences in normalized angular absorbance (Figure 4e), UBF outperforms PF by 12.5% per year on average. The photothermal advantage over CF is more pronounced. On average, PF yields 48.9% greater vapor production than CF, while UBF exceeds CF by 67.6% (Tables S6–S9). UBF provides a key advantage for regions with lower solar availability, where the sun spends the majority of the day and year at steep angles (exceeding 50°) rather than directly overhead. For example, in McCook, Nebraska, and Wuwei, China, UBF yields 144.86 and 151.44 L/yr, respectively, despite these regions having substantially lower solar availability than cities like Mokha or Cairo. PF also demonstrates highly competitive performance. In Mokha, Yemen, it yields nearly 168 L/yr (UBF: 188.30 L/yr). In McCook and Wuwei, PF produces 128.25 and 134.09 L/yr, respectively. In contrast, CF yields only 85.87 and 89.80 L/yr in those same cities, meaning PF outperforms CF by approximately 49.3%. As noted, these estimations are based on textile evaporators roughly the size of a man's hand. If scaled to a 1 m^2^ area (equivalent to 25 units), UBF is estimated to produce ∼4708 L/yr (Table S6). Furthermore, while the initial 20 × 20 cm textile weighs only ∼11.2 g, an entire 1 m^2^ would weigh just ∼281 g, which is lighter than a small 10 oz bottle of water.

Based on a preliminary materials cost analysis, the estimated cost is ∼$0.6216 per one 20 × 20 cm. This amount accounts for the procurement of raw materials (wool textile, dopamine HCl, and sodium periodate), as well as the water consumption and wastewater management (Table S10). Here, the calculation excludes the energy expenditures for water heating and the plasma treatment. As detailed in our previous study, these energy costs are approximately $0.0754 per 1 m^2^, with potential reductions dependent on the production batch size [50].

The combination of high volumetric yield, minimal mass and bulk, and low materials cost highlights the system's substantial portability and scalability, offering a highly viable solution for socially and economically vulnerable communities.

### 2.4 Outdoor Evaporation Performance With 10 wt.% NaCl Solution

**FIGURE 5:**
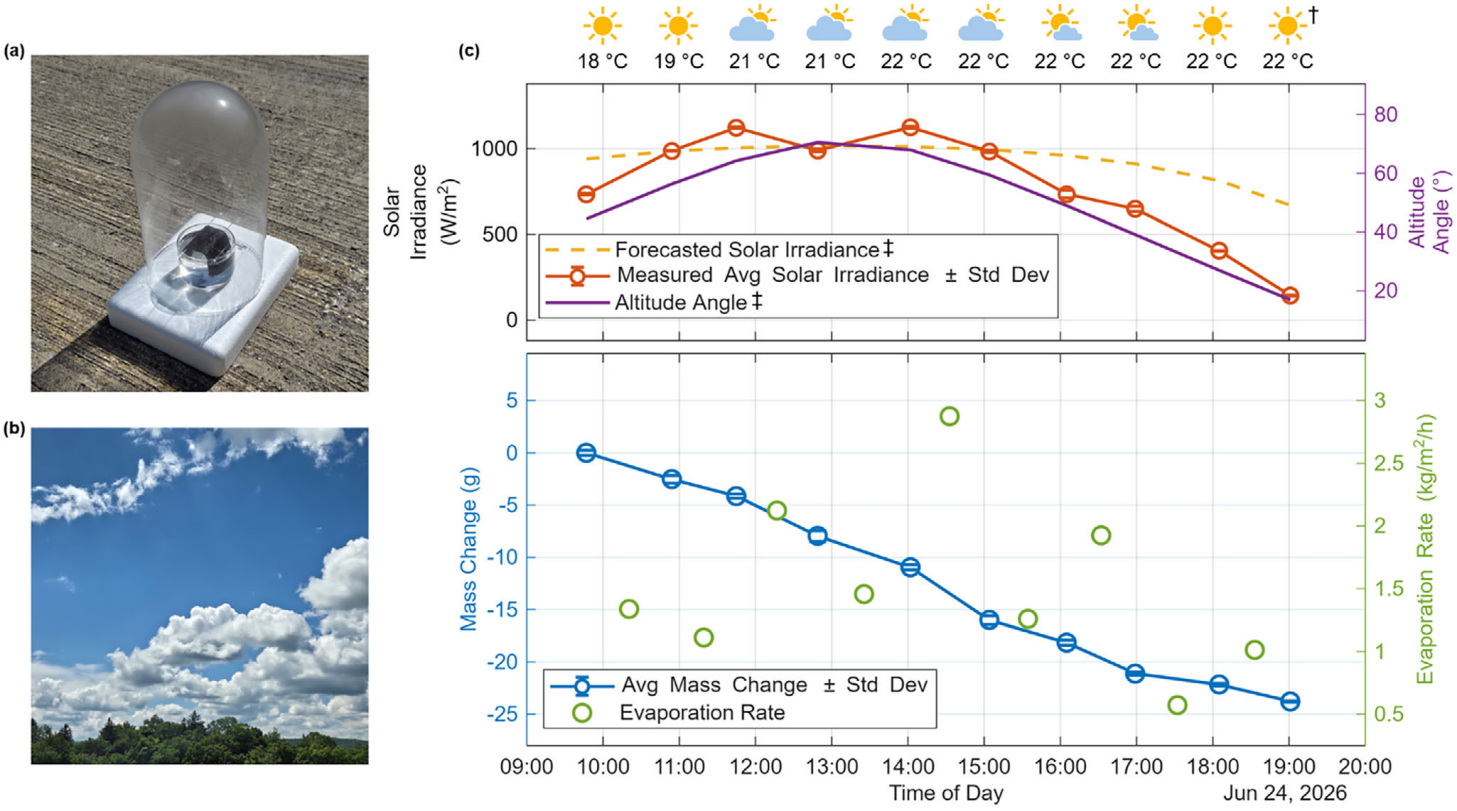
(a) Photograph of the experimental setup for demonstration of real‐world application using a UBF and 10 wt.% NaCl solution. (b) Photograph of the weather conditions for the outdoor experiment. (c) Comprehensive view of recorded outdoor experimental data. † Hour‐by‐hour weather data reproduced from timeanddate.com (Location: Ithaca, NY, USA) [69]. ‡ Forecasted Solar Irradiance and Altitude Angle data reproduced from suncalc.org (Location: 176 Hoy Road, Ithaca, NY, USA) [70].

To evaluate the evaporation capability of UBF, we use a high concentration (10 wt.%) NaCl solution (Figure 5). To simulate a practical use case, the textile is positioned horizontally without optical concentrators (Figure 5a). This configuration, as mentioned, exposes the top surface to incoming sunlight while leaving the underside open, facilitating simultaneous evaporation from both sides. Two metal racks are used to suspend the textile. The effective surface area used for the evaporation rate calculation is based on the textile surface exposed above the water, which was 1.75 in × 1.5 in (4.45 cm × 3.81 cm). Here, 1.75 in width and ∼1 in length of the textile is horizontally oriented while the remaining ∼0.5 in length of the textile curves over the two wires and hangs nearly vertically downward. A glass dome, the same that was used in the distillate analysis in Figure 3i, is placed over the setup to condense the evaporated vapor. Because engineering and optimizing the collection system is beyond the scope of this study, the dome primarily serves to simulate realistic, highly humid distillate‐collection conditions. As shown in Figure 5b, the weather during the experiment was mostly sunny with clouds passing throughout the day. The solar irradiance recorded (Extech SP505) is presented together with an imported database, which does not account for the clouds and other air pollution (Figure 5c).

We quantify the evaporation performance by monitoring the mass loss of the system. Between around 09:30 and 19:00, the UPF ISVG system evaporated approximately 24 g of the 10 wt.% NaCl solution (Figure 5c). This yields an average evaporation of 1.52 kg/m^2^/h, with a peak rate reaching 2.88 kg/m^2^/h. Given the partially cloudy weather, high‐salinity water, and simplified construction of this field test, these results demonstrate the significant potential of the PDA‐wool textile for a simple ISVG. Furthermore, no salt accumulation was observed during or after the experiment. Combined with the natural salt dissolution capability presented in Figure 2c, continuous salt rejection is expected throughout the day and night.

With the findings established in this study, future efforts will focus on translating this simple innovation into large‐scale applications. First, expanding the evaporation test over extended durations in extreme environments will further highlight the material's robust longevity. Second, since we validate our system in a less‐than‐ideal geographic location, a textile‐based ISVG system in a solar‐abundant, water‐scarce region presents an opportunity to quantify its maximum continuous yield under ideal use cases. Finally, leveraging the inherent scalability of PDA‐wool textiles to develop integrated, multi‐module arrays offers a clear pathway to optimize and maximize production rates for a broader impact.

## 3 Conclusions

In summary, we report a nature‐inspired, 2.5D polydopamine (PDA)‐wool textile as a high‐performing and sustainable evaporator for interfacial solar vapor generation (ISVG). Through the synergistic integration of natural wool's intrinsic moisture‐wicking and evaporative capabilities, PDA's broadband photothermal conversion, and plasma‐etched hierarchical structuring, this design achieves robust evaporation rates of 2.21 and 2.43 kg/m^2^/h under 1‐Sun illumination for PDA‐wool and ultrablack PDA‐wool, respectively. Crucially, this all‐natural system overcomes the material and fabrication limitations of conventional 3D evaporators by ensuring an easily scalable dyeing method, low‐cost material, salt‐free stability, all while eliminating the environmental risks associated with microplastics and nanomaterials. While this work lays the groundwork for PDA and ultrablack textiles as effective seawater ISVG, additional studies that include rain and wastewater in combination with enhanced water collection design could expand the potential of this material system. Ultimately, our eco‐friendly textile approach demonstrates significant potential for scalable deployment, providing practical, light, and safe solar‐driven water production technology options for resource‐scarce regions and decentralized global water management.

## 4 Experimental Section

### 4.1 Materials

Single jersey wool and double knit (interlock) wool textiles were purchased from Nature's Fabrics, PA, USA, and flannel wool textiles (#527 and #527 in black) were purchased from Testfabrics, PA, USA. Dopamine hydrochloride (≥98%) and sodium periodate (≥99.8%) were purchased from Sigma–Aldrich. Sodium chloride (NaCl) was purchased from VWR Chemicals, USA. Natural Seawater was purchased from Carolina Biological Supply, USA. The right‐angle silver mirrors were purchased from Edmund Optics, NJ, USA.

### 4.2 Fabrication of PDA‐Wool Textiles and the Subsequent Ultrablack Treatment

Deionized (DI) water was poured into 500 mL bottles and placed in a shaker (Thermo Scientific MaxQ 4450). Undyed wool fabrics were immersed in the water at a material‐to‐liquor ratio of 1:150 mL. The water was heated to 50°C with a shaking speed of 100 RPM. Once the water reached 50°C, dopamine hydrochloride (1 g/L) was added. After 10 min, sodium periodate (1 g/L) was added to the solution. After 20 min, the dyed wool fabrics were washed in a water bath and allowed to air‐dry at room temperature. Once dried, PDA‐wool textiles were placed in the plasma chamber (PE‐100, Plasma Etch, Inc.) and initiated at 40 W power for an 80‐min duration without any gas flow under 0.1 Torr vacuum.

### 4.3 Scanning Electron Microscopy

Scanning electron microscopy (SEM, Zeiss Gemini 500 FE‐SEM) was utilized for high‐magnification imaging of textiles. Accelerating voltage was 0.75–1 kV. Textiles were sputter‐coated with Au‐Pd for 60–80 s (Denton Desk V).

### 4.4 UV–vis–NIR Spectrophotometry

Total reflectance (%R) and total transmittance (%T) were measured with a UV–vis–NIR spectrophotometer (Agilent Cary 5000) with an integrating sphere (Agilent Cary External Diffuse Reflectance Accessory). The total absorbance (%A) was calculated from A = 1‐(R+T). The variable‐angle center‐mount accessory was used to measure the transflectance (%TR). Counterclockwise tilt represented the negative angle, and the clockwise tilt represented the positive angle, ranging from −60° to +60°.

### 4.5 Moisture Content and Moisture Retention

Textile samples were cut into 38 mm in diameter using a circular fabric cutter (SDL Atlas) and placed in the conditioning room with temperature set to 21°C ± 2°C (70 ± 4°F) with relative humidity of 65% ± 5% for a minimum of 8 h (ASTM D1776‐20). Then, the mass of samples at equilibrium (*E*) and the mass of samples after drying at 105°C for 1 h (*D*) were obtained to calculate the moisture content (MC) (ASTM D2654‐22):

(2) $$MC\;\left(\%\right)=100\times \frac{\left(E-D\right)}{E}$$

In the same conditioning room, another set of samples was used to measure the mass of samples at equilibrium (*W_1_*) and the mass of samples saturated with water (*W_2_*). *W_2_* of the samples was measured after submerging a sample in water for 1 min and vertically hanging for 5 min. The calculation of the moisture retention (MR) follows AATCC TM199‐2018e:

(3) $$MR\;\left(\%\right)=100\times \frac{W_{2}-W_{1}}{W_{1}}$$

### 4.6 Water Vapor Permeability

In the conditioning room (ASTM D1776‐20), 45 g of DI water was poured into a dedicated ∼82 cm diameter (∼32.25 inches diameter) dish to fill up to 10 ± 1 mm under the lip. On a mesh support, circular cut textile (larger than the diameter of the dish) was placed and were compressed with a circular metal frame that matched the diameter of the dish. Then the edge of the textile and the frame were taped together on the outer rim to fully enclose any potential openings. The mass of this assembly (*M_1_*) was measured and placed on the SDL Atlas M261 rotating table. After 24 h (*t*), the mass of the assembly (*M_2_*) was measured to calculate the water vapor permeability (WVP, g/m^2^/day) (British Standard 7209):

(4) $$WVP=\frac{24\left(M_{1}-M_{2}\right)}{At}\;$$

where *A* was the area of the exposed test fabric, which is equivalent to the internal cross‐sectional area of the dish.

### 4.7 Air Permeability

Textile samples were cut to ∼25.4 mm (1 inch) in diameter and mounted in the Capillary Flow Porometer (CFP‐1100‐AEHXL, Porous Materials, Inc.) and selected Frazier Analysis. The resulting data were presented in ft^3^/min/ft^2^ per 0.5 inches of water as an indication of the amount of pressure applied to the textile samples.

### 4.8 Salt Dissolution

UBD was prepared by cutting the undyed wool textile into 1.5‐inch by 3‐inch, and using the previously described dyeing and plasma etching methods. Two wires were placed parallel to each other across a beaker. The wires were spaced 1‐inches apart. The sample was placed on the wires, and 1‐inch of each side of the textile was overhung downward. Underneath, 135 g of 10 wt.% NaCl solution was added to saturate the sample through wicking. The wires and the elevated surface of the textile sample were not submerged in the water. 0.75 g of NaCl was spread over a 0.75‐inch by 1‐inch area in the center of the elevated surface of the sample.

### 4.9 Solar Vapor Generation Setup

1E: PDA‐wool textiles were cut to 28 mm by 70 mm. For UB samples, plasma etching was conducted after cutting the dyed textiles into this dimension. Two slits were made on the polystyrene sheet, each slit in 3 mm by 32.5 mm dimension. Both edges of the cut sample were pushed through downward to expose a 28 mm by 28 mm surface on top of the stage, and two edges (15 mm in length) were exposed to be partly submerged into the bulk seawater underneath. 2E: PDA‐wool textiles were cut to 32.5 mm by 50 mm. For UB samples, plasma etching was conducted after cutting the dyed textiles into these dimensions. An aluminum plate was waterjet cut to create two U‐shaped frames, allowing an exposure area of 26 ± 1 mm square, clamping the textile sample. Aluminum tape was used to enclose the two sides and the top of the clamped frame. Silver mirrors were placed to illuminate a 25 mm by 25 mm area within the frame. The bottom edge of the sample (15 mm in length) was partly submerged into the bulk seawater underneath through a slit (3 mm by 38 mm). A total of 215–220 mL of seawater was filled into the glassware to allow ∼5 mm between the stage and the water‐level. The glassware containing the bulk seawater was covered with aluminum foil, and the bottom surface of the stage was covered with aluminum foil. The location of the evaporation setup was maintained nearly identical for all experiments conducted. The evaporation setup was placed on a digital balance (Ohaus Scout). The 1 kW/m^2^ irradiance from the solar simulator (Newport Oriel Sol3A) was calibrated with a pyranometer (Hukseflux LP02) at the beginning of every experiment.

### 4.10 Evaporation Rate Calculation

All evaporation rates for the seawater, 1E, and 2E, for both dark and illuminated conditions were calculated using Equation (5):

(5) $$\dot{m}=\frac{\left|\frac{\Delta m}{\Delta t}\right|}{A}\;$$

where $\dot{m}$ is the evaporation rate in kg/m^2^/h, *Δm* and *Δt* are the change of mass (g) and change of time (s), respectively. The effective surface area (*A*) of the bulk seawater corresponded to the internal cross‐sectional area of the glassware, and $\dot{m}$ was measured without the cover stage. The *A* for the 1E and 2E setups were 784 mm^2^ (28 mm by 28 mm) and 625 mm^2^ (25 mm by 25 mm), respectively.

### 4.11 Salinity and Salt Rejection Ratio Calculation

Due to the salinity probe (Mettler Toledo SD30) being limited to producing two decimal places, an alternative conversion metric was used to calculate the salinity using Equation (6) [71]:

(6) S=0.000523σ

where S is the salinity in g/kg, and *σ* is the conductivity in µS/cm. The ratio of salt input and output was quantified using:

(7) $$R=\left(1-\frac{C_{d}}{C_{s}}\right)\;\times 100\%$$

where R is the salt rejection ratio, and *C_s_* and *C_d_* represent the salt concentrations of the starting seawater and the collected distillate, respectively.

## Author Contributions

K.P. and L.M.S. conceived the initial concept. L.M.S. and L.Z. supervised the project. K.P. designed and executed experiments. J.G. performed simulations, and K.P. and J.G. interpreted the simulation results. K.P., H.J., and M.B. conducted sample preparation and characterization experiments. Y.W. managed the solar simulator system and assisted with interpretation. K.P. analyzed and visualized results and wrote the manuscript. All authors contributed to the final manuscript.

## Conflicts of Interest

The authors declare the following competing interests: Patent Application: Cornell University. Inventors: Larissa M. Shepherd, Kyuin Park, and Hansadi Jayamaha. 11396‐01‐US. Filed Provisional Patent. The method and data of plasma treating wool to create an ultrablack textile are covered in the provisional patent and manuscript. The authors declare no other competing interests.

## Supporting information




**Supporting File 1**: advs77609‐sup‐0001‐SuppMat.docx.

**Supporting File 2**: advs77609‐sup‐0002‐S1.mp4.

## Data Availability

The data that support the findings of this study are available from the corresponding author upon reasonable request.
