# Supplementary material for: Polydopamine‐Wool Textiles for Sustainable Interfacial Solar Vapor Generation
Source: Adv Sci (Weinh). 2026 Sep 6:e77609. Online ahead of print. doi: 10.1002/advs.77609 (PMC13547712; doi:10.1002/advs.77609)
Supplement: Supplementary file 1 [file ADVS-9999-e77609-s001.docx]

Supporting Information

Polydopamine-Wool Textiles for Sustainable Interfacial Solar Vapor Generation

Kyuin Park, Jintong Gao, Hansadi Jayamaha, Yipu Wang, Mia Bressler, Lenan Zhang, Larissa M. Shepherd*

K. Park, H. Jayamaha, Prof. L. M. Shepherd

Department of Human Centered Design, Cornell Human Ecology

Cornell University, Ithaca, NY 14853, USA

E-mail: [larissa.shepherd@cornell.edu](mailto:larissa.shepherd@cornell.edu)

J. Gao, Y. Wang, Prof. L. Zhang

Sibley School of Mechanical and Aerospace Engineering, Cornell Duffield Engineering

Cornell University, Ithaca, NY 14853, USA

M. Bressler

School of Civil and Environmental Engineering, Cornell Duffield Engineering

Cornell University, Ithaca, NY 14853, USA

**Figure S1.** Large-scale PDA-dyeing of wool textiles. Photographs of the before and after dyeing, using ~15 cm width and ~400 cm length of single jersey wool textile.

**Figure S2.** Photograph of PDA-Wool textiles (PS, PD, and PF) on the top and Ultrablack wool textiles (UBS, UBD, and UBF) on the bottom row.

**Figure S3.** The total reflectance (%R) and the total transmittance (%T) data utilized to calculate the total absorbance (%A).

**Figure S4.** Photographs of white undyed wool textiles illustrate the inherent open structure of the textiles and the topography of their surfaces. Top image with WS, WD, and WF (left to right) on a ruler demonstrates the high-%T of both single jersey (S, left) and double knit (D, center). Bottom image with the same order of textiles exhibits the loose internal architecture of the D textile (center) and the napped hairy layer of fibers on top and bottom of the flannel (F) wool (right). Images taken under D65 standard lighting condition.

**Figure S5.** Infrared (IR) emissivity of WS, PS, and UBS. Measured using FT-IR with an integrating sphere (Bruker Vertex 80v with PIKE Technologies Mid-IR IntegratIR).

**Figure S6.** Photographs of time-dependent salt dissolution using UBD.

**Table S1.** Evaporation rate data for PS, PD, PF, UBS, UBD, and UBF in 1S1E, 1S2E, and 2S2E setups with the initial and final temperature readings of the bulk seawater and the surrounding ambient condition. Digital hygrometer (Extech 445814) was placed ~20 cm at same horizontal level to measure the relative humidity. Temperature and humidity vary between samples due to fluctuations in laboratory ventilation system and the weather conditions.

**Supplementary Note S1. (Table S2, Figure S7-S9)**

**Numerical simulations for textile solar evaporator**

A two-dimensional (2D) simulation model was constructed for the solar evaporation setup, including a textile solar evaporator and surrounding air (Figure S7). Natural wind was represented by an imposed airflow through the domain, while solar energy input was introduced as a boundary heat source on the evaporator surface. Multiple evaporator arrangements (including 1S1E, 1S2E, and 2S2E) were examined to compare their transport characteristics under different operating conditions. We performed numerical simulations by coupling three physics interfaces: laminar flow, heat transfer in fluids, and moisture transport in air. The airflow field was solved using the laminar flow interface under steady-state conditions, based on the continuity and Navier–Stokes equations for an incompressible fluid,

$\text{ρ}\text{(}\frac{\text{∂}\text{u}}{\text{∂}\text{t}}\text{ + }\text{u}\text{⋅∇}\text{u}\text{) = -}\text{∇}\text{p }\text{+ }\text{μ}\text{∇}^{\text{2}}\text{u}\text{ + }\text{ρ}\text{g}$	(1)

$\text{∇⋅}\text{u}\text{ }\text{= 0}$	(2)

where ***g*** is the gravitational acceleration. *p* is the pressure. *ρ* and *μ* represent density and dynamic viscosity of air, respectively. The resulting velocity field was then coupled with heat and moisture transport to describe convective heat transfer and vapor removal around the evaporator.

We modeled water vapor transport in the surrounding air using the moisture transport in air interface. The water vapor concentration field was governed by a convection–diffusion equation, in which water vapor was transported by the local airflow and molecular diffusion in air. The vapor flux was expressed as

$\text{M}_{\text{v}}\frac{\text{∂}\text{c}_{\text{v}}}{\text{∂}\text{t}}\text{ + }\text{∇⋅}\text{(}\text{M}_{\text{v}}\text{u}\text{c}_{\text{v}}\text{ + }\text{J}_{\text{v}}\text{)}\text{ = }\text{G}$	(3)

$\text{J}_{\text{v}}\text{ = -}\text{M}_{\text{v}}\text{D}\text{∇}\text{c}_{\text{v}}$	(4)

$\text{c}_{\text{v}}\text{ = }\text{ϕ}_{\text{w}}\text{c}_{\text{sat}}$	(5)

where *Μ*_v_ is the molar mass of water vapor. *c*_v_ represents the local vapor concentration. ***u*** is the velocity field. ***J***_v_ is the vapor flux. *G* is the moisture source term associated with evaporation at the interface. *D* is the vapor diffusion coefficient in air. *ϕ*_w_ is the relative humidity. *c*_sat_ is the vapor saturation concentration.

Heat transfer is modeled using the heat transfer in fluids interface. The temperature field was governed by the energy conservation equation,

$\text{ρ}\text{c}_{\text{p}}\text{(}\frac{\text{∂}\text{T}}{\text{∂}\text{t}}\text{ + }\text{u}\text{⋅∇}\text{T}\text{) = }\text{∇⋅}\text{(}\text{k}\text{∇}\text{T}\text{) + }\text{Q}$

where *c_p_*, *k*, and *T* are heat capacity, thermal conductivity, and temperature, respectively. *Q* is the volumetric heat source term. This equation accounts for both heat conduction and convective heat transport driven by the surrounding airflow. The airflow field obtained from the laminar flow simulation was coupled into the heat transfer model to describe convection-enhanced heat dissipation around the evaporator. As shown in Figure S6, several boundary conditions are applied in the simulation model to describe heat fluxes.

$\text{α}\text{q}_{\text{in}}\text{ = }\text{q}_{\text{conv}} \text{+} \text{q}_{\text{eva}}$

$\text{q}_{\text{conv}}\text{ = (}\text{T }\text{- }\text{T}_{\text{a}}\text{)}\text{h}_{\text{a}}$

$\text{q}_{\text{eva}}\text{ = }\text{J}_{\text{v}}\text{h}_{\text{fg}}$

*q*_in_ is the solar energy flux applied to the evaporator surface. *q*_conv_ and *q*_eva_ represent the heat flux of convection and evaporation. The parameters *α*, *h*_a_, and *h*_fg_ represent spectrally averaged solar absorptance, heat transfer coefficient, and effective evaporation enthalpy, respectively. *T*_a_ is ambient temperature. The vapor flux *J*_v_ was calculated based on the local temperature at the evaporation surface.

**Table S2. Input parameters used in the numerical simulation**

| Parameter | Value | Unit | Description |
| --- | --- | --- | --- |
| *L* | 0.2 | m | Length of air domain |
| *W* | 0.15 | m | Width of air domain |
| *H* | 0.15 | m | Height of air domain |
| *L*_eva_ | 0.025 | m | Length of evaporator |
| *H*_eva_ | 0.0007 | m | Height of evaporator |
| *u*_air_ | 0.06–0.2 | m s^-1^ | Flow velocity of air |
| *q*_in_ | 500–3000 | W m^-2^ | Input energy flux |
| *T*_air_ | 25 | ºC | Ambient temperature |
| RH | 0.15 |  | Relative humidity |
| *ρ*_air_ | 1.184 | kg m^-3^ | Density of air |
| *μ*_air_ | 1.85×10^-5^ | Pa s | Dynamic viscosity of air |
| *k*_air_ | 0.0257 | W m^-1^ K^-1^ | Thermal conductivity of air |
| *D* | 2.6×10^-5^ | m^2^ s^-1^ | Diffusion coefficient of water vapor in air |
| *α* | 0.7179–0.9554 |  | Solar absorptance |
| *h*_fg_ | 2357 | kJ kg^-1^ | Evaporation enthalpy of water |

**Figure S7.** Simulation domain and boundary conditions for textile solar evaporator. The textile evaporator is modeled in the 1E (a, one evaporation surface) and 2E (b, two evaporation surfaces) configuration, respectively. The surrounding air domain has dimensions of 0.2 × 0.15 × 0.15 m. Air (orange arrows) flows through the domain with a velocity of *u*_air_ to represent natural wind.

Several boundary conditions (red arrows) are applied to the evaporator to account for input energy flux (*q*_in_), convective heat loss (*q*_conv_), and evaporative heat loss (*q*_eva_). In the 1E configuration, the evaporator is placed horizontally, with solar irradiation incident vertically on its top surface. In the 2E configuration, the evaporator is placed vertically, and solar irradiation is applied either to one side (one-sided illumination and two evaporation surfaces, 1S2E) or to both sides (two-sided illumination and two evaporation surfaces, 2S2E).

**Figure S8. Mesh-independence study.** The evaporation rate was simulated using progressively refined meshes containing 6292, 24610, 57718, 101215, 232401, and 640257 elements, respectively. The predicted evaporation rate gradually converged as the number of mesh elements increased. Mesh 5 was selected for simulations to balance accuracy and computational cost.

**Figure S9**. a) a-e) Simulated evaporation profiles of the UBF sample under varying illumination and structural configurations: (a) dark 1E, (b) dark 2E, (c) 1-Sun 1E (1S1E), (d) 1-Sun 2E (1S2E), and 2-Sun 2E (2S2E). f) Effect of convection on the 1E and 2E setups during dark evaporation. g) Effect of increasing ambient temperature on the evaporation rate of UBF across all configurations. h) Effect of varying convection on the evaporation rate for the flannel (F) samples across all configurations. i) Effect of increasing total solar energy on the evaporation rate of F samples on the 2E setup.

**Table S3.** Evaporation rate data for CF, PF, and UBF in 1S1E, 1S2E, and 2S2E setups with the initial and final temperature readings of the bulk seawater and the surrounding ambient condition. Digital hygrometer (Extech 445814) was placed ~20 cm at same horizontal level to measure the relative humidity. Temperature and humidity vary between samples due to fluctuations in laboratory ventilation system and the weather conditions.

**Figure S10.** Long-term evaporation rate stability test using UBF in 1S2E configuration.

**Supplementary Note S2. (Figure S11, S12, Table S4, S5)**

**Color Change and Durability Assessment of Flannel Wool Textiles**

Light fastness testing was conducted according to AATCC TM16.3 using a xenon arc lamp light fastness tester (TESTEX TF420). Blue reference fabric served as the control. All fabric samples were exposed to light for 42 hours at relative humidity of 30 RH% and ambient temperature at 40°C. Such accelerated condition, relative to UV spectrum in AM1.5G solar irradiance, is equivalent to approximately 9 to 11 days with 4-5 h/day of continuous peak sunlight. Light fastness was evaluated for undyed flannel wool (WF), PDA-dyed flannel wool (PF), and ultrablack flannel wool (UBF), with results reported as the average of three separate samples.

Visual assessment of the textiles was conducted with CIELAB (X-Rite Ci7800). L* is the lightness value (0 to 100), a* is green/red value (-100 to +100), and b* is blue/yellow value (-100 to +100) (Figure S11 and Table S4).

Tensile strength and extension properties were evaluated using an Instron tensile tester (Instron 5566) following a modified ASTM D5034-09 protocol. A 50 mm gauge length was used, with an extension rate of 100 mm/min applied along the warp direction. Fabric swatches measuring 50 mm × 75 mm were used to allow for xenon arc lamp UV exposure prior to tensile testing. Tensile strength was measured for WF, PF, and UBF, for before and after UV exposure. Results are reported as the average of three separate samples (Figure S12 and Table S5). All fabric samples were conditioned for a minimum of 8 hours prior to testing in accordance with the standard.

**Figure S11.** Lightness (L*) comparison of flannel wool textiles for before and after accelerated UV exposure. Raw data in Table S2.

**Table S4.** CIELAB (L*, a*, and b*) visual assessment on WF, PF, and UBF for before and after accelerated UV exposure.

**Figure S12.** Tensile strength and extension properties of WF, PF, and UBF for before and after accelerated UV exposure. Raw data in Table S3.

**Table S5.** Durability assessment on WF, PF, and UBF for before and after accelerated UV exposure.

**Figure S13.** Visual similarities of commercially black-dyed flannel wool (CF) on left and PDA-dyed flannel wool (PF) on right under D65 standard lighting.

**Figure S14.** Plotted illustration of UBF’s and CF’s shift in solar-weighted absorbance with respect to solar incidence angle (Lambert’s cosine law) using normalized absorbance, derived from the transflectance measurement. Superimposed plot on the right shows the difference in the level of absorbance between UBF and CF when incident angle is tilted by 60°.

**Table S6**. Calculated sum of estimated annual vapor production utilized to plot Figure 4g (20 x 20 cm). Table for 1m^2^ for scaled-up scenario, equivalent to 25 units.

**Table S7**. Example raw data (Day 1, 24-hr) for estimated vapor production using CF’s normalized solar absorbance and evaporation rate. Annual water production column presents the total sum of 365 days’ hourly evaporation rate column.

**Table S8**. Example raw data (Day 1, 24-hr) for estimated vapor production using PF’s normalized solar absorbance and evaporation rate. Annual water production column presents the total sum of 365 days’ hourly evaporation rate column.

**Table S9**. Example raw data (Day 1, 24-hr) for estimated vapor production using UBF’s normalized solar absorbance and evaporation rate. Annual water production column presents the total sum of 365 days’ hourly evaporation rate column.

|  | 20cm x 20cm | unit | $ | Material cost | Source |
| --- | --- | --- | --- | --- | --- |
| Wool fabric 280gsm | 11.2 | g | 0.6127 | $3163.50 per 206.52 m^2^ | https://www.onlinefabricstore.com/rk-classics-chatman-wool-jersey-ivory-fabric-.htm |
| Water | 1.68 | L | 0.0059 | $13.36 per 1000 gallons | https://www.epa.gov/watersense/data-and-information-used-watersense |
| Dopamine HCl | 1.68 | g | 0.0013 | $750 per 1000 kg | https://www.chemicalbook.com/ProductDetail_EN_dopamine-hydrochloride_2920079.htm |
| Sodium Periodate | 1.68 | g | 0.0017 | $1000 per 1000 kg | https://www.chemicalbook.com/ProductDetail_EN_1232630.htm |
| **Total** |  |  | **0.6216** |  |  |

**Table S10.** Materials cost analysis. Estimation for bulk order of wool, dopamine hydrochloride, and sodium periodate and combined cost of industrial water consumption and wastewater production.
